# GPU-accelerated Monte Carlo based scatter correction in brain PET/MR

**DOI:** 10.1186/2197-7364-1-S1-A32

**Published:** 2014-07-29

**Authors:** Michaela Gaens, Julien Bert, Uwe Pietrzyk, A Autret, N Jon Shah, Dimitris Visvikis

**Affiliations:** Institute of Neuroscience and Medicine - 4, Forschungszentrum Juelich, Kragujevac, Germany; LaTIM, INSERM, UMR1101, CHRU, Brest, France; Department of Mathematics and Natural Science, University of Wuppertal, Kragujevac, Germany

To develop and evaluate a fast and accurate Monte Carlo simulation (MCS) based scatter correction for PET/MR imaging, based on Geant4 using graphical processing units (GPUs).

The GPU implementation by Bert et al. [1] including the photon tracking within a voxelized volume was extended with the modeling of the detection process in the Siemens MR-BrainPET. Using NVIDIA’s CUDA photon pairs undergo several kernel functions in parallel, from their generation according to the activity distribution up to their detection in the scanner. Subsequently, coincidences are sorted and stored in list-mode. The new GPU implementation (GeForce GTX690) was validated by comparison with Gate v6.2 on a single CPU (Intel Core i7, 3.4GHz), showing a speedup factor of 135. The use of multiple GPUs can further increase this acceleration.

For Compton scatter correction the reconstructed emission image, the patient MR-derived attenuation map and a CT-derived attenuation template of the MR coils are used as input for the simulation. The resulting simulated projection data are scaled to the normalized measured projection data. Thus, the often unstable fit to the distribution tails, necessary for single scatter simulation (SSS)[2], is circumvented. The scaled simulated scatter distribution is then incorporated in the reconstruction. The proposed scatter correction methodology was evaluated and compared to the SSS approach using sphere phantom measurements. Finally, the effect on patient data was investigated.

Phantom results suggest an improved contrast with our new MC-based scatter correction, especially for measurements including the MR coils, where the contrast recovery was improved by 10-14%. First patient images also show improved grey/white matter contrast.

**Table 1:** Comparison of recovery coefficients after scatter correction based on MCS and SSS. MC-based scatter correction shows improved recovery coefficients for the hot spheres.

Recovery Coefficient [%]	27 mm cold	22 mm cold	17 mm hot	13 mm hot	10 mm hot	8 mm hot
MCS	82.5	79.3	95.4	85.0	71.6	80.0
SSS	81.8	77.8	80.6	72.1	60.5	65.9
Difference	0.7	1.5	14.8	12.9	11.1	14.1

**Figure 1:**
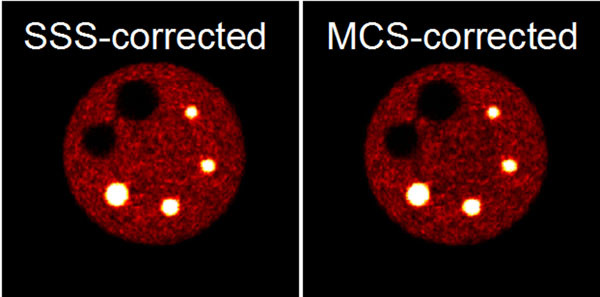
Assessment of image quality according to NEMA NU 2-2007. Images show the phantom with a hot spheres/background ratio of 8:1 measured with MR head coils inside the BrainPET scanner.

**Figure 2:**
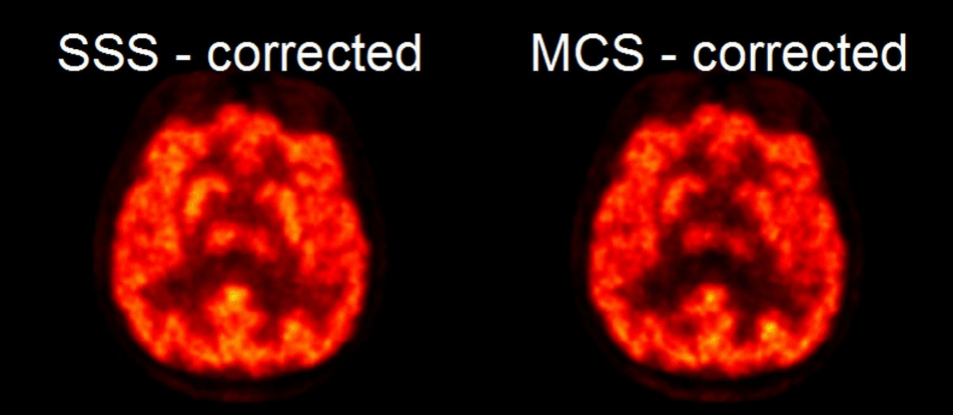
Comparison of reconstructed FDG images using the scatter estimate provided by the vendor’s SSS (left) and our MCS method (right). Images were reconstructed using the vendor provided 3D OP-OSEM algorithm with 2 subsets and 32 iterations.

The new GPU-accelerated MCS allows for fast and accurate estimation of scattered coincidences in PET studies using the Siemens MR-BrainPET with a major speed-up compared to standard MCS and superior image quality compared with the standard correction based on SSS.
